# The Public Attitude Towards Selecting Dental Health Centers

**Published:** 2014-09

**Authors:** Vahid Moshkelgosha, Mehrdad Mehrzadi, Ali Golkari

**Affiliations:** a Orthodontics Research Center and Dept. of Orthodontics, School of Dentistry, Shiraz University of Medical Sciences, Shiraz, Iran.; b Students’ Research Committee, School of Dentistry, International Branch, Shiraz University of Medical Sciences, Shiraz, Iran.; c Dept. of Dental Public Health, School of Dentistry, Shiraz University of Medical Sciences, Shiraz, Iran.

**Keywords:** Dentists, Oral health, Health services, Attitude to health

## Abstract

**Statement of the Problem:** No published literature was found studying the people's reasons on why to choose or not to choose a dental care setting in south Iran, while understanding their attitude towards choosing their dental care center is consequential for planning a successful oral health care service system.

**Purpose:** To determine the factors affecting how people of the city of Shiraz choose their dental health services.

**Materials and Method:** A cross-sectional analytic study was designed. A self-administered questionnaire was produced, tested and then distributed among 570 multistage randomly selected parents of schoolchildren of the city of Shiraz. Independent t-test, paired t-test and Spearman correlation were used to analyze the factors influencing participants in choosing clinics for their esthetic and non-esthetic dental treatments.

**Results:** 400 questionnaires were complete and analyzed. The recommendation from others was found to be the most encouraging factor to choose a dentist or a dental clinic. More importance was reported for various factors affecting participants' choice of dental clinic when seeking non-esthetic treatments, while recommendation and reputation of dentist/dental clinic played a vital role in esthetic treatments. The cost was more important for respondents living in more deprived districts (*p*= 0.05), for unemployed group (*p*< 0.001) and for those with less education (*p*< 0.001).

**Conclusion:** Factors affecting people's choice for dental care proved to be highly complicated. Recommendation was found playing an important role. Dental patients consider various factors when looking for non-esthetic treatment but would go for the best possible when seeking esthetic treatments. Findings of this study indicate that patients’ choice and utilization of dental service can be improved if dental clinics provide high quality of dental care with reasonable fees.

## Introduction

The impact of oral diseases is significant on both individuals and communities. The reasons that oral diseases are considered important public health problems include, but not limit to, their high prevalence in almost every society, their heavy effects on people's quality of life, and their cost of treatment in terms of both finance and time [1]. It is true that dental diseases have been reduced over the last decade in most developed countries. However, first, this decline has not been seen in most developing countries; and second the cost of dental services has been increased. Therefore, governments are in continuous challenge to upgrade their oral health care delivery system to improve their people's oral health. Understanding when people seek dental services, how they choose their services, what they expect from such facilities, and what prevents them from expressing their needs, are vital factors for planning a good oral health care system.

Studies have shown that regular dental attendance leads to better oral health outcomes and improves people's quality of life [2]. However, Individuals’ utilization of dental services depends on a variety of factors, including access to care, financial problems, and attitudes toward dental care. Socioeconomic [3-4], racial [5], and age [6] variables affect access to dental health services. These factors, in turn, may vary across geographic locations and demographic groups [7].

Patients’ concepts of oral health care services are important and have a potential impact on the utilization of dental care and satisfaction with the results of using such services [8]. Few studies have investigated the concerns and views of populations towards health care services in different parts of the world [9-11]. Today, patients’ perceptions, concepts, and satisfaction with dental health care services are recognized as main measures of the quality assurance programs. Information collected through patients’ surveys has proven to be a successful way of strategic evaluation and improving the quality of health services [12].

Although research is done routinely to evaluate different aspects of marketing in almost every field, only a few scientific papers have been published trying to evaluate factors affecting utilization of health services. In developing countries, such as Iran, health services are usually planned based solely on the professionals' opinions, ignoring the lay people's attitudes, wants, and cultural differences. No evidence was found in the literature on how dental services are distributed in Iran, and on how Iranians choose them. Therefore, this study was set to determine the factors affecting the residents of the city of Shiraz in choosing their dental health services: factors they like about a dentists and factors that stop them from going to one. Shiraz is the largest city in Southern Iran. The southern half of Iran is generally less affluent and has less dentists/dental settings than the northern half. About 60% of the population in the south of Iran is urban. Many dental patients from a wide area in this part of country are referred to Shiraz for general and specialized dental treatments. 

## Materials and Method

A cross-sectional study was designed. Parents of pupils of schools of the city of Shiraz (in south of Iran) were chosen as the study population for two reasons: First, the parents of schoolchildren were considered mature and financially independent people who make their own decision on where to go seeking dental treatments, while younger Iranian adults might still be under their parents' influence. Second, in Iran, the type of school the children go is a reliable way to understand the socioeconomic status of the family. This way the authors were able to select good numbers of students from public and private types of school, invite their parents into the study and divide them into two socio-economic groups based on the type of their children's schools. 

A sample size of about 405 people was calculated to achieve the objectives of the study. Estimating a 70% response rate, 570 questionnaires were distributed among parents of schoolchildren using multistage random sampling. Eight schools were randomly selected in collaboration with the Shiraz education bureau from the four educational zones (one public and one private school from each zone) of the city of Shiraz. The number of students selected in each school was based on the number of the students enrolled in public and private schools of each zone. The required number of schoolchildren in each school was selected randomly using the school register. A letter was sent to the parents of each selected child explaining the objectives and process of the study. They were assured about their privacy and confidentiality of the data they would provide. Authors' office number was provided in case they needed more information. A self administered questionnaire was attached. They were asked to fill the questionnaire only if they were happy to participate.

Related literature was used by authors to find appropriate questions [8-16] and then develop a questionnaire in Farsi. The content and face validity of the questionnaire were evaluated by five dental consultants and a Farsi editor (all faculty members of Shiraz University of Medical Sciences). Necessary corrections were made to the questions based on their opinion. Twenty conveniently selected adult volunteers were invited to answer the questions in a pilot study. They were asked if they had any difficulty reading, understanding or answering the questions. Corrections were made to the questions if necessary. The final questionnaire consisted of three parts: the first part included Socio-demographic variables such as age, sex, and educational level. The second part included the questions about frequency of use of oral health care services, type of the dental clinic visited, the reason for the last dental visit and factors determining their decision for hesitating to seek dental advice and treatment at a regular basis. In the third part of the questionnaire, the participants were asked about factors determining their decision for choosing a dental clinic for different types of dental services (esthetic and non-esthetic). 

The collected data were entered and statistically analyzed by SPSS 18. Descriptive statistics were used to delineate the study population. Spearman correlation was used to assess the similarities of answers related to esthetic and non-esthetics treatments. Independent and paired t tests were used to assess the differences between answers of participants with different backgrounds and between their answers related to esthetic and non-esthetic treatment needs. The significant level was set at 0.05.

## Results

Of the 570 distributed questionnaires, 415 questionnaires were returned. A further 15 questionnaires were not filled properly and so were excluded from the study. Therefore, 400 questionnaires (response rate = 70.2%) were included in the final analysis. Characteristics of the study population are shown in Table 1.

**Table 1:** Characteristics of the study population (N= 400).

**Variable **	**Group **	**Number **	**Percentage**
Age	<30 yrs	95	23.75
	30-40 yrs	232	58.00
	40-50 yrs	68	17.00
	>50 yrs	5	1.25
Sex	Female	297	74.25
	Male	103	25.75
Education level	Up to high school	273	68.25
	University first degree	101	25.25
	Postgraduate degree	26	6.50
Employment status	Housekeeper/unemployed	237	59.25
	Average income job	148	37.00
	High income job	15	3.75
Residential district^ a^	Affluent	176	44.00
	Deprived	224	56.00
Child school	Public	259	62.75
	Private	149	37.25
Dental visit history	None in last 2 years	100	25.00
	Once in last 2 years	124	31.00
	>once in last 2 years	176	44.00
Reason for the last dental visit ^b^	Esthetic	11	3.18
	Non-Esthetic	318	91.91
	Both/routine check up	17	4.91
Type of dental clinic they usually go	State	205	51.25
	Private	180	45.00
	Could be any	15	3.75

a. Based on schools' neighborhood. b. Some respondents did not answer all the questions. c. More than one reason could be selected.

Nearly 75% of the participants visited the dentist at least once during the last 2 years, with less than 10% seeking esthetic treatments. About half of them said they usually attend private dental clinics. 

The participants were asked about the most important factors encouraging them for choosing a dental clinic. They were asked to say, on a five-point Likert scale, how important is each factor when they seek esthetic and non-esthetic dental treatments. Their answer to the four most chosen factors (short distance from home, low tariffs, recommendation by a friend or relative and convenient appointment times) is shown in Table 2.

**Table 2:** Frequency of answers to questions about encouraging factors for selecting a dental clinic (N=400)

**Encouraging factor**	**N (%)**				
This factor is important for me when choosing a dental clinic for …	Fully agree	Partially agree	No opinion	Partially disagree	Fully disagree
For esthetic treatments					
Short distance from home	67(16.8)	101(25.3)	87(21.8)	104(26)	41(10.3)
Low tariffs	154(38.5)	148(37)	46(11.5)	42(10.5)	10(2.5)
Recommended by friends or relatives	155(38.8)	193(48.3)	42(10.5)	6(1.5)	4(1)
Convenient appointment times	144(36)	157(39.3)	51(12.8)	35(8.8)	13(3.3)
For non-esthetic treatments					
Short distance from home	88(22)	116(29)	84(21)	84(21)	28(7)
Low tariffs	173(43.3)	137(34.3)	37(9.3)	42(10.5)	11(2.8)
Recommendation by friends or relatives	165(41.3)	189(47.3)	33(8.3)	10(2.5)	3(0.8)
Convenient appointment times	158(39.5)	167(41.8)	37(9.3)	32(8)	6(1.5)

The "recommendation from a friend or relative" was the most important factor in both esthetic and non-esthetic dental care. It followed by "low tariffs" and "convenient appointment times" which were almost equally important. The "short distance of clinic from home" attracted less importance.

There was a significant correlation in participants' answers to how important is each factor between answers related to esthetic and non-esthetic treatments (*p*< 0.001, r= 0.772). However, in general, they illustrated more importance for factors affecting them in choosing their clinic for non-esthetic dental treatments (*p*< 0.001). The difference of importance score given to the distance of clinic from home with scores given to other factors was markedly larger when asked about esthetic treatments than non-esthetic treatments (*p*< 0.001). This finding indicates that participants were more likely to travel to a farther clinic when they were seeking dental esthetic treatment.

No statistically significant difference was found in answers given to questions between the two sexes (*p*= 0.756) or among participants of different age groups (*p*= 0.589). In both esthetic and non-esthetic treatments, the cost of dental care was more important for respondents living in more deprived districts than those in more affluent areas (*p*= 0.049). Distance and convenient appointment times were also more important for those living in more deprived districts, but only when seeking esthetic dental services. On the contrary, reputation of dentist/dental clinic was more important for those from more affluent areas (*p*= 0.029).

The employment status also had an effect on how respondents answered the questions. For those who were unemployed or housekeeper, the low cost of dental care was more encouraging than the others in both types of dental care (*p*< 0.001). The importance given to "low tariffs" and "short distance" was reversely correlated with participants' education level (*p*< 0.001; r= 0.677), indicating those with higher education were more likely to pay more or to travel more to get the service from whom they trusted more.

When asked about the most important factor stopping or discouraging them from going to a specific dental clinic, "high tariffs" was mentioned by more than half (54.75%) of the participants. So the cost of treatments was markedly more important than any other reason for not choosing a dental clinic (Table 3).

**Table 3:** Reasons discouraging the participants for selecting a dental clinic (N=400)

**Variable**	**N (%)**
Cost of dental care	219(54.75)
Long distance from home	14(3.50)
Poor quality of treatment	92(23.00)
Hygienic concerns	26(6.50)
Poor reputation	22(5.50)
Unfavorable working hours	2(0.50)
Others	25(6.30)

## Discussion

This study was conducted to determine the factors affecting Iranian patients' decision on where to go for esthetic and non-esthetic dental treatments, and to compare the importance of those factors. Various factors were mentioned by participants rather than those that were specifically cited by researchers. The results of this research showed that the most important encouraging factor for choosing a dental care service was the recommendation by friends or relatives. Other more important factors were cost of delivered care and convenient treatment appointment. 

The outcome of the dental treatment depends largely on the quality of dental care received. It could be predicted based on the previous studies [12-14] that patients' satisfaction would be vital to attract other patients to a dental clinic. Patients usually seek for the dental care services which has good quality, utilizing modern technologies and done by competent dentists (treatment quality and competence of operators are often used synonymously) and recommend it to their friends and relatives.

The cost of dental care is found to be an important factor affecting people's choice of dental clinic. Considering the economic situation in which Iranian people live in, and the wide inequality of resources available to people of different backgrounds, the cost of treatment proved to mainly affect those of lower socio-economic backgrounds. This finding was also in accordance with the findings of the previous studies [15-16].

In addition, persons with lower education have more concerns about dental care cost. This may be a reflection of the fact that the people with lower education often end up unemployed and financially more stressed. This finding necessitates the importance of reviewing the cost of dental services in dental clinics to make treatment fees more affordable to public.

Several studies reported that understandable communication and friendly behavior of dental team are important factors for patients with direct effect on utilization patterns [17]. Difficulty in getting an appointment and long waiting lists, which was found important in this study, especially when seeking non-esthetic treatments, were also found to be among the most issues causing dissatisfaction in other studies [18-20].

The distance of the dental clinic from participants’ homes, which was found to have less impact on people's decision in the current study, might be of more concern to people living in larger cities (compared to people living in Shiraz). It is assumed that the larger the city is (and thus with crowded streets and more traffic jams), the more important the distance factors becomes in encouraging the persons in choosing a dental clinic. This explains the controversial findings of some other studies [12] in which the distance was found the more important effect. Of course, the cultural differences might have an important effect.

Furthermore, the distance might have larger impact for those living where more options are available to them (compared to those who travel from other towns/cities to Shiraz). Another study conducted in Tunzania also found that people living in rural areas or where health services are difficult to access, have to travel a long distance anyway. Therefore, they think less of the distance they should travel when deciding between two or more clinics [21].

The latter justification is specifically true when people think of where to go for esthetic treatments. People found to be more tolerable of long distances in the current study when seek esthetic dentistry. This finding might be due to the fact that the professionals providing esthetic dental services are scarce in the study region and people are willing to accept some difficulties in order to use such services.

The current study evaluates the important perceived factors in utilization of dental health services among Shiraz citizens. Responding to these needs and concerns may help further improvement in community oral health conditions and proper use of dental services available. Understanding and predicting the patients’ behavior regarding dental health services is necessary for planning such services in every community and the purpose of this research is to evaluate the questions raised in this field. Considering the nature of the practical use such study might have for oral health service policy makers, this study was conducted on a relatively small sample that were limited to just one city. The authors propose further similar investigation in sub urban and rural part of country to complement the current data. 

## Conclusion

Factors affecting people's choice for dental care proved to be highly complicated. Recommendations, especially from friends and relatives, were found playing an important role. It seems that most dental patients in the target population would go for the best possible dentist they know when seeking esthetic treatment, while they would think more of several other factors such as distance and costs when they need non-esthetic dental care. It was also shown that dental patients have little problem with their chosen dentists' working hours or long waiting lists, and are ready to adapt themselves with their desired dentists' available appointment timing. 
